# Acute mesenteric ischemia diagnosed using the aquarium sign: A case report

**DOI:** 10.1097/MD.0000000000033735

**Published:** 2023-05-12

**Authors:** Ushio Higashijima, Motohiro Sekino, Naoya Iwasaki, Hiroshi Araki, Tetsufumi Motokawa, Yusuke Inoue, Yasuhiro Taniguchi, Shinya Sato, Yasushi Miyazaki, Tetsuya Hara

**Affiliations:** a Department of Anesthesiology and Intensive Care Medicine, Nagasaki University Graduate School of Biomedical Sciences, Sakamoto, Nagasaki, Japan; b Department of Cardiovascular Medicine, Nagasaki University Hospital, Sakamoto, Nagasaki, Japan; c Department of Surgery, Nagasaki University Graduate School of Biomedical Sciences, Sakamoto, Nagasaki, Japan; d Department of Gastroenterology and Hepatology, Nagasaki University Graduate School of Biomedical Sciences, Sakamoto, Nagasaki, Japan; e Department of Hematology, Nagasaki University Hospital, Sakamoto, Nagasaki, Japan; f Department of Hematology, Atomic Bomb Disease and Hibakusha Medicine Unit, Atomic Bomb Disease Institute, Nagasaki University, Sakamoto, Nagasaki, Japan.

**Keywords:** aquarium sign, critically ill patients, echocardiography, intensive care, intestinal ischemia, point-of-care ultrasound

## Abstract

The diagnosis of mesenteric ischemia in critically ill patients remains challenging; however, the aquarium sign, comprising a large number of bubble images in the right cardiac chambers on echocardiography, may be used as a point-of-care ultrasound finding to diagnose acute mesenteric ischemia (AMI).

**Patient concerns:**

A 65-year-old woman diagnosed with lymphoma was urgently admitted to the intensive care unit with suspected tumor lysis syndrome. High-dose vasopressor and inotropic agents were required to manage the patient’s shock with marked lactic acidosis and peripheral hypoperfusion with mottled skin, and multidisciplinary treatment was initiated. By day 6, the lactate levels normalized and there were no abnormal abdominal findings. An echocardiogram was performed to examine the mass lesion associated with lymphoma in the right atrium and evaluate the hemodynamics; it revealed an “aquarium sign.” Similar findings were found in the inferior vena cava and portal vein.

**Diagnoses:**

Contrast-enhanced computed tomography of the abdomen revealed hepatic portal vein gas, poor contrast of the colon wall, and intramural emphysema, and a diagnosis of AMI was made. Lower gastrointestinal endoscopy showed necrosis of the colon.

**Interventions:**

The patient underwent urgent subtotal colorectal resection.

**Outcomes:**

Although a tracheostomy was required, the patient’s general condition improved after surgery, and she was discharged to the ward without mechanical ventilatory support in the intensive care unit on Day 19.

**Lessons:**

In patients with risk factors for AMI, repeated evaluation for the presence of aquarium signs by echocardiography may be warranted, even if there are no abdominal findings or abnormalities in biomarkers, such as lactate levels and trends. When the aquarium sign is found, AMI should be aggressively suspected, and a definitive diagnosis should be made to initiate early therapeutic intervention.

## 1. Introduction

Acute mesenteric ischemia (AMI), represented by non-occlusive mesenteric ischemia (NOMI), is one of the major causes of death in critically ill patients admitted to the intensive care unit (ICU).^[1,2]^ AMI occurring in the ICU was reported to result in a 58% mortality rate^[3]^; therefore, early diagnosis is important to improve prognosis. However, there are no specific symptoms or biomarkers for the diagnosis of AMI,^[4–6]^ and the opportunity for more invasive testing, such as contrast-enhanced computed tomography (CT), to confirm the diagnosis is easily lost. Thus, diagnosis of AMI in critically ill patients remains challenging.

The aquarium sign is a rare finding that presents as a large number of bubbles seen in the cardiac chambers on echocardiography, which resemble bubbles in an aquarium.^[7]^ In recent years, the aquarium sign in the right cardiac chambers has attracted much attention as a finding indicating AMI in critically ill ICU patients.^[8,9]^ The presence of the aquarium sign can be easily and noninvasively repeatedly assessed using bedside echocardiography. Therefore, the aquarium sign may be a useful point-of-care ultrasound (POCUS) finding to diagnose AMI.

Here, we report a case in which AMI was not suspected from abdominal findings and biomarkers, such as lactate levels, but was suspected based on the aquarium sign. Emergency surgery was performed after a definitive diagnosis was made by contrast-enhanced CT and gastroendoscopy.

## 2. Case report

A 65-year-old woman (height, 148 cm; weight, 45 kg) was diagnosed with diffuse large B-cell lymphoma and hospitalized for chemotherapy. The patient’s medical history included adult Still disease, sarcoidosis, Sjogren syndrome, mucosa-associated lymphoid tissue lymphoma, and rheumatoid arthritis, and her prescribed medications included salazosulfapyridine and anti-inflammatory analgesics for rheumatoid arthritis.

We observed a sudden deterioration in the patient’s general condition with disturbance of consciousness, tachypnea, and tachycardia. The patient had not undergone chemotherapy yet; however, she was suspected of having developed tumor lysis syndrome and was admitted urgently to the ICU. On admission to the ICU (ICU Day 1), the patient was in a state of shock with marked lactic acidosis (pH, 7.026; base excess, −19.5 mEq/L; lactate, 8.4 mmol/L) and peripheral hypoperfusion with mottled skin (Table 1). Therefore, multidisciplinary treatment was initiated, including invasive respiratory support, circulatory support, blood purification therapy, chemotherapy (adriamycin, cyclophosphamide, and methylprednisolone) for B-cell lymphoma, and antibiotic therapy (cefepime, vancomycin, and micafungin). She required aggressive fluid resuscitation (24-hour fluid balance, +4974 mL) and high doses of vasopressor and inotropic agents (noradrenaline, 0.5 μg/kg/min; vasopressin, 1.8 units/h; and adrenaline, 0.1 μg/kg/min).

**Table 1:** The patient’s laboratory data and findings from physical examination and echocardiography after intensive care unit admission.

Variables	ICU day						
	1	2	3	4	5	6	7
pH	7.026	7.392	7.414	7.439	7.490	7.350	7.392
Base excess (mmol/L)	−19.5	−1.5	1.1	3.0	0.2	−2.0	0.3
Lactate (mmol/L)	8.4	22.0	13.2	3.2	2.4	1.1	2.1
AST (IU/L)	43	10,178	16,460	11,473	6209	2111	848
LDH (IU/dL)	1271	12,230	14,665	7017	1230	493	298
D-dimer (μg/mL)	6.1	22.8	32.6	49.1	102.0	36.7	28.7
WBC (×10^3^/μL)	18.3	14.3	8.0	7.6	13.6	7.2	0.3
Abdominal distension	−	+	−	−	−	−	−
Peripheral hypoperfusion	+++	++	++	+	−	−	−
Aquarium sign	−	−	−	−	−	+++	−

AST = aspartate aminotransferase, ICU = intensive care unit, LDH = lactate dehydrogenase, WBC = white blood cells.

On ICU Day 2, the patient showed a trend toward recovery based on her hemodynamic state. Adrenaline was tapered and the dose of noradrenaline and vasopressin was also reduced. On the same day, the mottled skin disappeared; however, peripheral hypoperfusion of the fingers and toes persisted until ICU Day 4. Additionally, lactate and liver enzyme levels peaked on ICU Day 3, then declined (Table 1). The patient had abdominal distension on ICU Day 2, which improved the next day. Echocardiography was performed daily to follow up on the mass lesion associated with diffuse large B-cell lymphoma in the right atrium and evaluate the patient’s hemodynamics. Cardiac function showed normal cardiac contractility (ejection fraction, 63%) and no other abnormalities on ICU Day 2.

**Figure 1.**
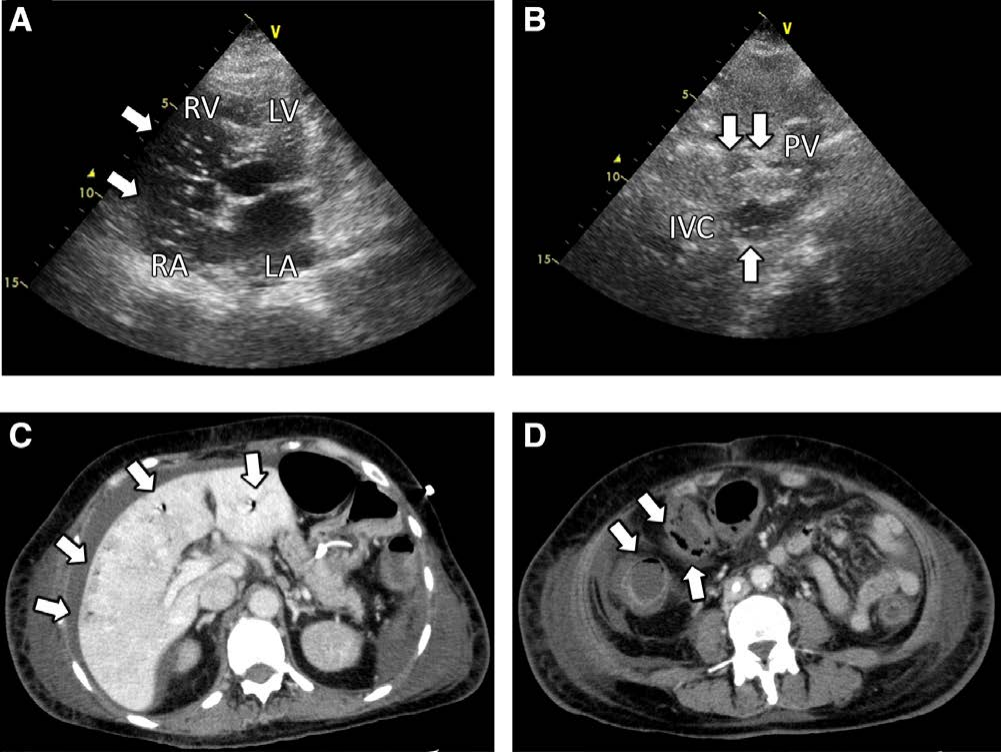
(1A, white arrows) A large number of bubbles were observed flowing from the right atrium towards the right ventricle. (1B, white arrows) Similar echogenic images were observed flowing in the portal vein and inferior vena cava. (1C, white arrows) Contrast-enhanced computed tomography of the abdomen was performed and revealed gas in the intrahepatic portal vein, as well as (1D, white arrows) poor contrast and intramural emphysema in the colon wall. IVC = inferior vena cava, LA = left atrium, LV = left ventricle, PV = portal vein, RA = right atrium, RV = right ventricle.

On ICU Day 6, the hemodynamics had stabilized (noradrenaline, 0.04 µg/kg/min) and ventilator weaning was planned. Moreover, lactate levels normalized, other nonspecific biomarkers known to show abnormal values during AMI showed a decreasing trend, and there were no abnormal abdominal findings on examination (Table 1). Echocardiography was performed to follow up on the mass lesion and hemodynamics, and a large number of bubbles was observed flowing from the right atrium toward the right ventricle (Fig. 1A, and see Supplementary Video S1, Supplemental Digital Content, http://links.lww.com/MD/I965, which demonstrated a large number of bubbles flowing in the right cardiac chambers). The patient was not receiving any fluids from either catheter that would have caused an iatrogenic air injection into the vein. Additionally, similar echogenic images were observed flowing in the portal vein and inferior vena cava (Fig. 1B, and see Supplementary Video S2, Supplemental Digital Content, http://links.lww.com/MD/I966, which demonstrated a large number of bubbles flowing in the portal vein and inferior vena cava). Suspecting AMI, contrast-enhanced CT of the abdomen was performed which revealed gas in the intrahepatic portal vein (Fig. 1C), as well as poor contrast and intramural emphysema in the colon wall (Fig. 1D). Additionally, lower gastrointestinal endoscopy revealed necrosis of the colon. Therefore, a subtotal colorectal resection was emergently performed. Immediately after the surgery, another echocardiography was performed, and the aquarium sign had disappeared.

Although a tracheostomy was required, the patient’s general condition improved, and she was discharged to the ward without mechanical ventilatory support on ICU Day 19. However, the patient died on the 20th day after ICU discharge due to multiple organ failure triggered by a new infection.

## 3. Discussion

The diagnosis of AMI in critically ill ICU patients remains challenging due to a lack of specific symptoms, biomarkers, and other simple, noninvasive diagnostic methods,^[4–6]^ leading to high mortality rates.^[2]^ In recent years, the aquarium sign, which can be evaluated using bedside echocardiography, has attracted attention as a pivotal POCUS finding for the diagnosis of AMI in an ICU setting.^[8,9]^ In the present case, we did not suspect AMI from the abdominal findings and biomarker values and trends. Therefore, if we had not suspected AMI based on the aquarium sign, the diagnosis may have been significantly delayed.

The aquarium sign is caused by hepatic portal venous gas (HPVG) flowing into the right atrium and ventricle, and AMI is one of the most critical differential diagnoses for HPVG.^[10]^ Although HPVG is caused by various factors, including inflammation of the gastrointestinal tract, obstruction and dilation, ulcers, malignancy, sepsis, iatrogenic injury, chemotherapy, and trauma, AMI accounts for the majority of HPVG (approximately 40–70%).^[10–13]^ AMI injures the mucosal barrier and, in association with excessive distension of intestinal loops and proliferation of gas-forming bacteria, gas migrates from the intestinal lumen to the mesenteric vein and through it to the portal vein and hepatic parenchyma.^[13]^ Previously, the mortality rate was reported to be 75%^[10,13]^; however, a recent Japanese database study reported this to be 27%,^[12]^ although the data was not limited to the ICU setting. This improvement in mortality may be attributed to the high utilization of CT, resulting in early diagnosis and intervention.^[12]^ Therefore, early diagnosis may lead to an improved prognosis.

AMI, represented by NOMI in the ICU, is often difficult to suspect from an interview or abdominal findings because many patients are sedated and there is a lack of specific symptoms.^[14]^ Abnormal acid–base balance, lactate, aspartate aminotransferase, lactate dehydrogenase, and D-dimer levels, as well as leukocytosis, are commonly used to suspect AMI; however, they do not identify the presence or absence of AMI with sufficient accuracy.^[4,5,14]^ Diagnosis is particularly difficult in critically ill patients because these values can be altered by factors other than AMI. As observed in the present case, hematologic malignancies can cause severe hyperlactatemia^[15]^ and other abnormalities, such as elevated aspartate aminotransferase, lactate dehydrogenase, and D-dimer levels, which would eliminate the opportunity to suspect AMI. In addition, in the present case, despite the presence of intestinal necrosis requiring surgical resection, the lactate level was normal on the day the diagnosis was confirmed, and other biomarkers showed a trend toward improvement.

In the ICU, where AMI can be masked, the aquarium sign may be a useful POCUS finding because it is noninvasive, can be easily and repeatedly assessed at bedside, and can lead to a definitive diagnosis by contrast-enhanced CT or gastrointestinal endoscopy, or in some cases, diagnostic laparoscopy.^[6]^ However, the aquarium sign is a rare finding; therefore, the pretest probability should be increased. Reported risk factors for NOMI include advanced age, history of heart failure, use of cardiopulmonary bypass, renal or hepatic dysfunction, and hemodialysis.^[4,6]^ In addition, high doses of vasopressors, as observed in this case, may reduce intestinal blood flow and cause NOMI.^[4,6]^ Furthermore, mottled skin is a sign of vasoconstriction and peripheral hypoperfusion and is also an early sign of possible AMI development in critically ill patients, including postoperative cardiac surgery patients.^[16–18]^ In addition to mottled skin, there are reports that intestinal blood flow correlates with capillary refill time, an indicator of peripheral hypoperfusion.^[19]^ The patient in the present report presented with severe and persistent peripheral hypoperfusion with mottled skin and was at high risk of developing AMI. A previous case was reported in which a patient with mottled skin presented with an aquarium sign, leading to a diagnosis of AMI.^[20]^ Critically ill patients with peripheral hypoperfusion are at high risk of developing AMI; therefore, it is important to continuously check for the aquarium sign, in addition to abdominal findings and biomarkers (along with the recognition of their limitations). Furthermore, it was reported that contrast-enhanced CT used for definitive diagnosis misses approximately 20 to 30% of AMI.^[2,21]^ When AMI is suspected based on various clinical findings but cannot be detected by contrast-enhanced CT and the patient is left for observation, it may be important to follow up on the clinical findings and repeatedly check for the presence of the aquarium sign.

The aquarium sign may be an important finding in the diagnosis of AMI in critically ill patients. Repeated evaluation may help detect AMI, especially in cases with risk factors for AMI. However, the frequency of occurrence of the aquarium sign in patients with AMI, as well as its sensitivity and specificity in diagnosis, are unknown. Further case accumulation and analysis are needed.

In conclusion, the diagnosis of AMI in critically ill patients remains challenging. In patients with risk factors for AMI, repeated evaluation for the presence of aquarium sign by echocardiography may be necessary. When the aquarium sign is found, AMI should be aggressively suspected, and a definitive diagnosis should be made to initiate early therapeutic intervention.

## Acknowledgments

We would like to thank Editage (www.editage.jp) for their English language editing.

## Author contributions

**Conceptualization:** Ushio Higashijima, Motohiro Sekino, Naoya Iwasaki, Hiroshi Araki, Tetsufumi Motokawa, Yusuke Inoue, Shinya Sato, Tetsuya Hara.

**Data curation:** Ushio Higashijima.

**Formal analysis:** Ushio Higashijima, Motohiro Sekino, Naoya Iwasaki, Hiroshi Araki, Tetsufumi Motokawa, Yusuke Inoue, Yasuhiro Taniguchi, Shinya Sato, Yasushi Miyazaki, Tetsuya Hara.

**Investigation:** Ushio Higashijima, Motohiro Sekino, Naoya Iwasaki, Hiroshi Araki, Tetsufumi Motokawa, Yusuke Inoue, Yasuhiro Taniguchi, Shinya Sato, Yasushi Miyazaki, Tetsuya Hara.

**Supervision:** Motohiro Sekino, Yasushi Miyazaki, Tetsuya Hara.

**Visualization:** Ushio Higashijima.

**Writing – original draft:** Ushio Higashijima.**Writing– review & editing:** Motohiro Sekino, Naoya Iwasaki, Hiroshi Araki, Tetsufumi Motokawa, Yusuke Inoue, Yasuhiro Taniguchi, Shinya Sato, Yasushi Miyazaki, Tetsuya Hara.

## Supplementary Material




